# Supplementary material for: High-Throughput Sequencing Reveals Differential Begomovirus Species Diversity in Non-Cultivated Plants in Northern-Pacific Mexico
Source: Viruses. 2019 Jun 29;11(7):594. doi: 10.3390/v11070594 (PMC6669537; doi:10.3390/v11070594)
Supplement: Supplementary file 1 [file viruses-11-00594-s001.pdf]

High-Throughput Sequencing Reveals Differential Begomovirus Species Diversity in Non-Cultivated Plants in Northern-Pacific Mexico

Supplementary Figure S1. Non-cultivated plants of the main plant families collected in natural ecosystem in regions of north-pacific Mexico. *Malvaceae*: A, *Malva parviflora.* D, *Abutilon trisulcatum.* J, *Malvastrum coronomandelianum.* K, *Herisantia crispa.* G, *Sida acuta.* M, *Sida rombifolia*. *Solanaceae*: B, *Nicotiana glauca.* E, *Nicotiana plumbanginifolia.* I, *Solanum trydinamum*. N, *Solanum rostratum*. *Asteraceae:* C, *Helianthus niveus.* F, *Sonchus oleraceus.* O, *Helianthus annus*. *Cucurbitacea:* L, *Momardica charantia*. *Fabaceae:* H, *Rhynchosia minima*.

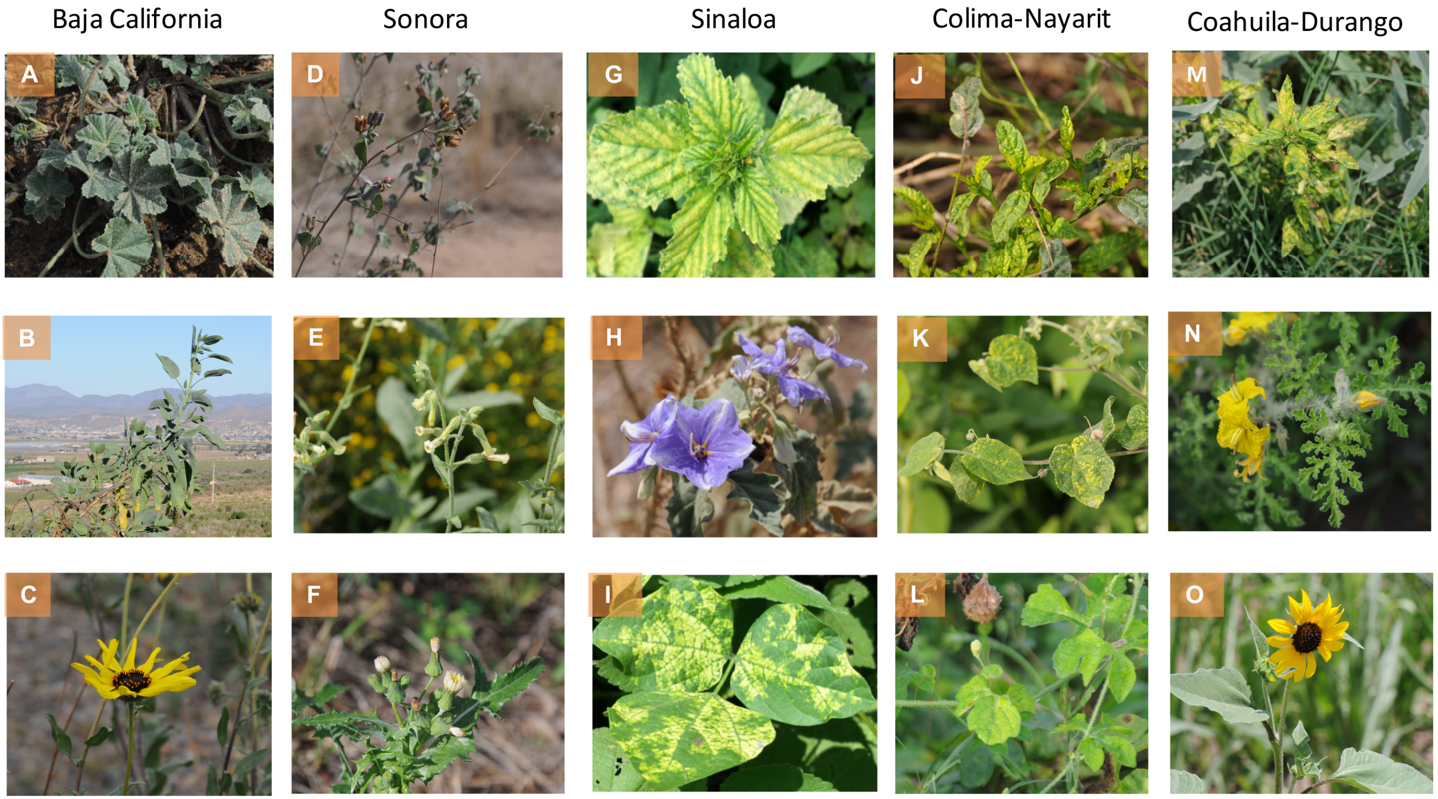

Supplementary Figure S2. Generation of Geminivirus-related signatures workflow**.** 1) Metagenomic reads obtained from each library were trimmed, 2) Each library was filtered (ViromeScan) for human, bacteria, and plant reads to obtain viral reads. 3) All filtered libraries were *de novo* assembled using SPAdes, and contigs were compared against the GeneBank non-redundant database using BLASTn for annotation. 4) Geminivirus-related signatures were classified, and sorted by contig length.

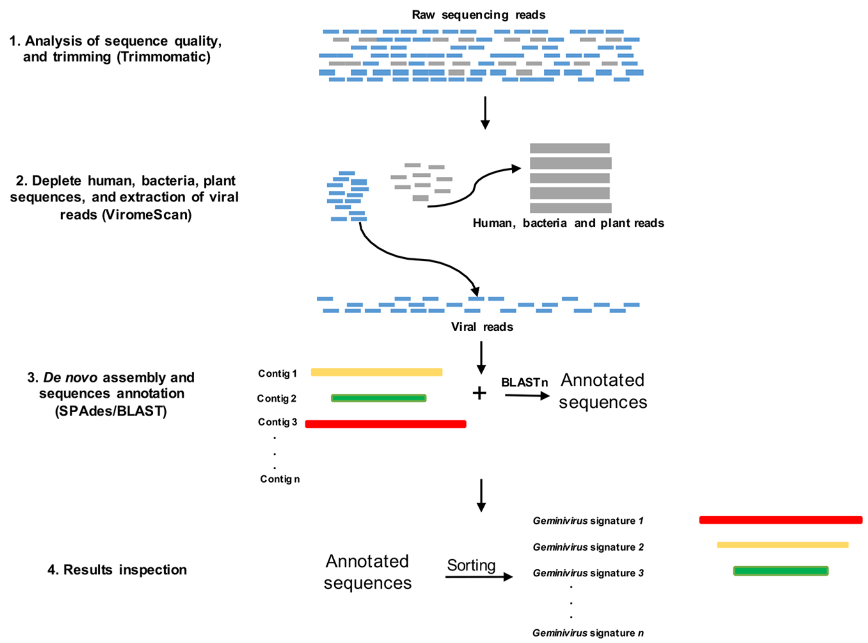

Supplementary Figure S3. PCR detection of five geminivirus species in three predominant plant families of non-cultivated plants from northern-pacific Mexico. PCR specific primers (Supplementary Table 6) were designed to detect *Pepper huasteco yellow vein virus* (PHYVV), *Tomato yellow leaf curl virus* (TYLCV), *Sida mosaic Sinaloa virus* (SiMSV), and *Rhynchosia golden mosaic virus*/*Rhynchosia golden mosaic Sinaloa virus* (RHGMV/RhGMSV). Plants from the *Fabaceae*, *Malvaceae*, and *Solanaceae* plant families sampled in five regions (Baja California, Sonora, Sinaloa, Colima/Nayarit, and Coahuila/Durango) were used (see Materials and Methods). A total of 126 specimens were analyzed for the presence of specific geminiviruses by PCR detection. The plant family and the scientific name of the plant species are indicated. Number of plant specimens analyzed by sampling area is indicated in parenthesis. Asterisks indicate the samples used for full-length viral genomes cloning (TYLCV from *N. glauca*, RhGMV and RhGMSV from *R. minima*, and SiMSV from *S. acuta*).

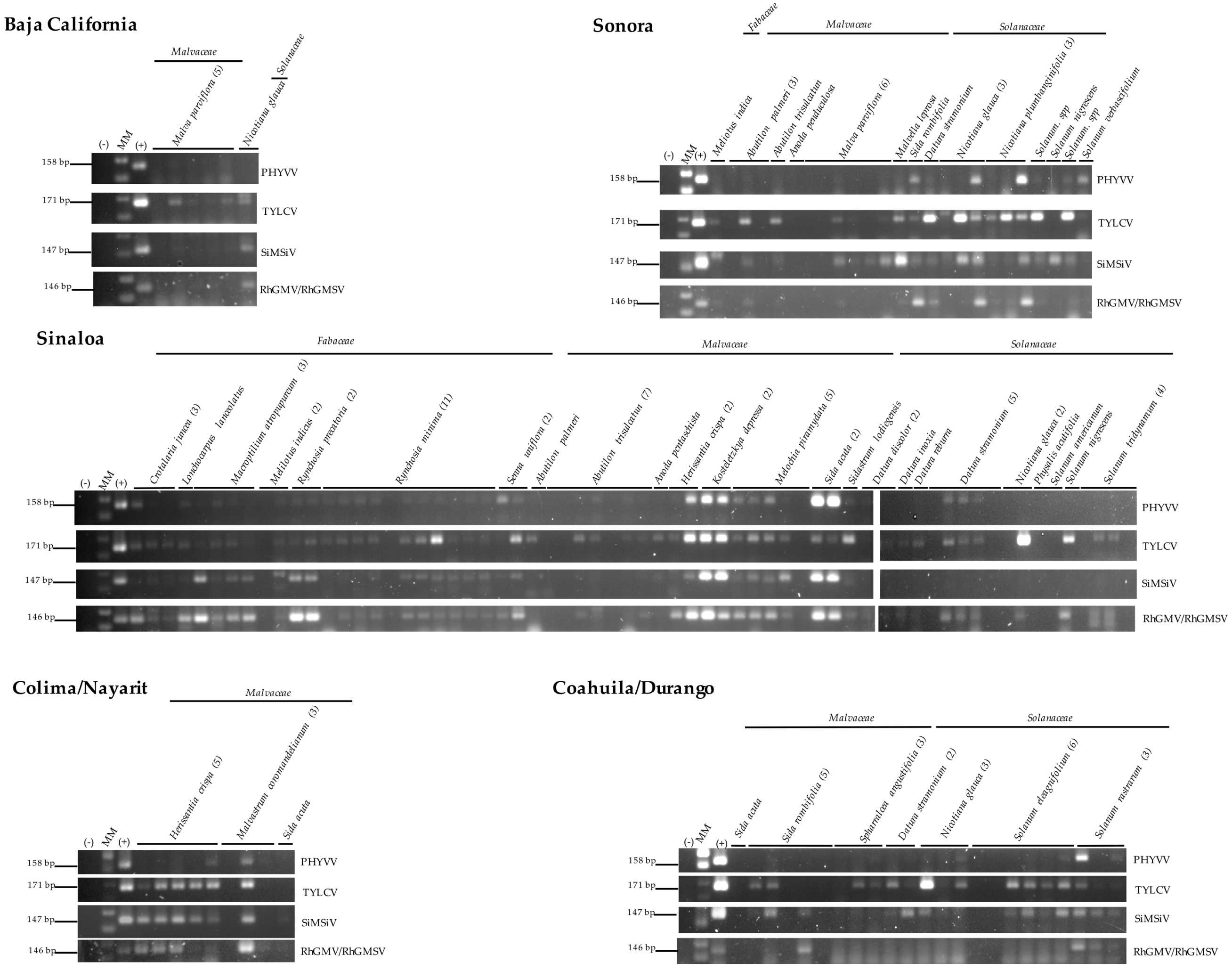

(-): PCR negative control (non-template); MM: molecular marker 1 Kb (ThermoFisher, UK); (+): PCR positive control (plasmidic DNA containing DNA-A of the respective geminivirus). Expected amplicon sizes are indicated by a small line.

Supplementary Table S1. Non-cultivated plants collected from northern-pacific regions of Mexico and determination of begomovirus host by PCR-test.

Sampling area[1]	Collection year	Plant Family	Plant species	Positive to begomovirus[2] (PCR test with universal primers)	Geolocalization
BC-EN	2015	*Asteraceae*	*Encelia farinosa*	+	31º 40′53.5″ N, 116º37′02.7″ W
BC-SQ	2014	*Asteraceae*	*Encelia farinosa*		30º26'0.596" N, 115º53'6.018" W
BC-SQ	2014	*Asteraceae*	*Erigeron spp*		30º26'0.596" N, 115º53'6.018" W
BC-EN	2015	*Asteraceae*	*Gnaphalium americanum*		31º 40′53.5″ N, 116º37′02.7″ W
BC-EN	2015	*Asteraceae*	*Helianthus niveus*	+	31º 40′53.5″ N, 116º37′02.7″ W
BC-EN	2015	*Asteraceae*	*Helianthus niveus*		31º 46′17.8″ N, 116º33′12.1″ W
BC-SQ	2015	*Asteraceae*	*Helianthus niveus*	+	30º34′31.8″ N, 115º52′50.3″W
BC-SQ	2015	*Asteraceae*	*Isocoma acradenia*		30º34′31.8″ N, 115º52′50.3″W
BC-SQ	2014	*Asteraceae*	*Sonchus oleraceus*	+	30º26'0.596" N, 115º53'6.018" W
BC-SQ	2015	*Asteraceae*	*Sonchus oleraceus*		30º30′57.1″ N, 115º59′30.0″W
BC-SQ	2014	*Boraginaceae*	*Heliotropium curassavicum*		30º26'0.596" N, 115º53'6.018" W
BC-SQ	2015	*Brassicaceae*	*Brassica tournefortii*	+	30º34′31.8″ N, 115º52′50.3″W
BC-EN	2015	*Brassicaceae*	*Raphanus raphanistrum*		31º 46′17.8″ N, 116º33′12.1″ W
BC-SQ	2015	*Chenopodiaceae*	*Chenopodium berlandieri*	+	30º30′57.1″ N, 115º59′30.0″W
BC-SQ	2015	*Chenopodiaceae*	*Chenopodium berlandieri*	+	30º32′04.1″ N, 115º57′13.5″W
BC-ME	2015	*Chenopodiaceae*	*Chenopodium spp*	+	32º24′42.5″ N, 115º06′53.5″ W
BC-SQ	2015	*Chenopodiaceae*	*Chenopodium spp*	+	30º32′04.1″ N, 115º57′13.5″W
BC-ME	2015	*Convolvulaceae*	*Convolvulus arvensis*		32º19′53.2″ N, 115º04′20.6″ W
BC-SQ	2014	*Convolvulaceae*	*Convolvulus arvensis*		30º26'0.596" N, 115º53'6.018" W
BC-EN	2015	*Cucurbitaceae*	*Marah macrocarpa*		31º 40′53.5″ N, 116º37′02.7″ W
BC-EN	2015	*Malvaceae*	*Malva parviflora*	+	31º 40′53.5″ N, 116º37′02.7″ W
BC-SQ	2015	*Malvaceae*	*Malva parviflora*	+	30º30′57.1″ N, 115º59′30.0″W
BC-SQ	2015	*Malvaceae*	*Malva parviflora*	+	30º30′57.1″ N, 115º59′30.0″W
BC-SQ	2015	*Malvaceae*	*Malva parviflora*	+	30º32′04.1″ N, 115º57′13.5″W
BC-SQ	2015	*Malvaceae*	*Malva parviflora*	+	30º32′04.1″ N, 115º57′13.5″W
BC-SQ	2015	*Malvaceae*	*Sphaeralcea ambigua*		30º30′57.1″ N, 115º59′30.0″W
BC-EN	2015	*Polygonaceae*	*Rumex crispo*		31º 40′53.5″ N, 116º37′02.7″ W
BC-ME	2015	*Polygonaceae*	*Rumex crispo*	+	32º20′06.8″ N, 115º04′13.2″ W
BC-SQ	2014	*Primulaceae*	*Anagallis arvensis*	+	30º26'0.596" N, 115º53'6.018" W
BC-SQ	2014	*Solanaceae*	*Datura stramonium*		30º26'0.596" N, 115º53'6.018" W
BC-EN	2015	*Solanaceae*	*Nicotiana glauca*	+	31º 40′53.5″ N, 116º37′02.7″ W
SO-HU	2015	*Amaranthaceae*	*Amaranthus palmeri*	+	26º 24′54.5″N, 109º01′15.1″W
SO-OB	2015	*Amaranthaceae*	*Amaranthus palmeri*	+	27º 36′22.5″N, 110º08′02.5″W
SO-NA	2015	*Amaranthaceae*	*Amaranthus spinosus*		26º96′00.39′′ N, 109º58 24.36′′ W

SO-HU	2015	*Amaranthaceae*	*Amaranthus spp*	+	26º54′97.04′′ N, 109º11′88.03′′ W
SO-HU	2015	*Amaranthaceae*	*Amaranthus spp*	+	26º 24′54.5″N, 109º01′15.1″W
SO-OB	2015	*Apiaceae*	*Conium maculatum*	+	27º 36′22.5″N, 110º08′02.5″W
SO-HU	2015	*Asteraceae*	*Ambrosia ambrosoides*	+	26º 24′54.5″N, 109º01′15.1″W
SO-OB	2015	*Asteraceae*	*Ambrosia ambrosoides*	+	27º33′33.5″ N, 110º05′17.6″ W
SO-HU	2015	*Asteraceae*	*Ambrosia cordifolia*		26º 24′54.5″N, 109º01′15.1″W
SO-HU	2015	*Asteraceae*	*Artemisia absitium*		26º 24′54.5″N, 109º01′15.1″W
SO-OB	2015	*Asteraceae*	*Artemisia ludoviciana*	+	27º33′33.5″ N, 110º05′17.6″ W
SO-OB	2015	*Asteraceae*	*Gnaphalium spp*	+	27º 08′09.9″N, 109º53′31.7″W
SO-OB	2015	*Asteraceae*	*Helenium mexicanum*	+	27º 36′22.5″N, 110º08′02.5″W
SO-NA	2015	*Asteraceae*	*Helianthus annuus*	+	26º 86′ 23.43′′N, 109º68′63.83 W
SO-NA	2015	*Asteraceae*	*Helianthus spp*		26º 86′ 23.43′′N, 109º68′63.83 W
SO-HU	2015	*Asteraceae*	*Perityle microglossa*		25º48'42.44" N, 108º13'9.302" W
SO-HU	2015	*Asteraceae*	*Perityle spp*	+	26º50'05.9"N, 109º31'51.8"
SO-HU	2015	*Asteraceae*	*Perityle spp*	+	26º50'05.9"N, 109º31'51.8"
SO-HU	2015	*Asteraceae*	*Sonchus oleraceus*		26º 24′54.5″N, 109º01′15.1″W
SO-NA	2015	*Asteraceae*	*Sonchus oleraceus*		26º 86′ 23.43′′N, 109º68′63.83 W
SO-NA	2015	*Asteraceae*	*Sonchus oleraceus*		26º 86′ 23.43′′N, 109º68′63.83 W
SO-NA	2015	*Asteraceae*	*Sonchus oleraceus*		26º96′00.39′′ N, 109º58 24.36′′ W
SO-HU	2015	*Bignoniaceae*	*Handroanthus impetiginosus*		26º 24′54.5″N, 109º01′15.1″W
SO-HU	2015	*Boraginaceae*	*Heliotropium curassavicum*	+	26º47′06.1″N, 109º40′52.8″W
SO-NA	2015	*Boraginaceae*	*Heliotropium curassavicum*		26º90′05.02′′N, 109º52′28.42′′ W
SO-NA	2015	*Boraginaceae*	*Heliotropium curassavicum*	+	26º90′05.02′′N, 109º52′28.42′′ W
SO-NA	2015	*Boraginaceae*	*Heliotropium curassavicum*	+	26º90′05.02′′N, 109º52′28.42′′ W
SO-OB	2015	*Boraginaceae*	*Heliotropium curassavicum*	+	27º 08′09.9″N, 109º53′31.7″W
SO-HU	2015	*Brassicaceae*	*Brassica juncea*		26º 24′54.5″N, 109º01′15.1″W
SO-RC	2015	*Brassicaceae*	*Diplotaxis muralis*		32º25′07.8″ N, 114º49′34.7″ W
SO-NA	2015	*Chenopodiaceae*	*Chenopodium album*	+	26º90′05.02′′N, 109º52′28.42′′ W
SO-NA	2015	*Chenopodiaceae*	*Chenopodium album*		26º90′05.02′′N, 109º52′28.42′′ W
SO-NA	2015	*Chenopodiaceae*	*Chenopodium album*	+	26º 86′ 23.43′′N, 109º68′63.83 W
SO-NA	2015	*Chenopodiaceae*	*Chenopodium album*	+	26º96′00.39′′N, 109º58′24.36′′ W
SO-NA	2015	*Chenopodiaceae*	*Chenopodium album*		26º 86′ 23.43′′N, 109º68′63.83 W
SO-OB	2015	*Chenopodiaceae*	*Chenopodium berlandieri*		27º 08′09.9″N, 109º53′31.7″W
SO-OB	2015	*Chenopodiaceae*	*Chenopodium berlandieri*	+	27º 08′09.9″N, 109º53′31.7″W
SO-RC	2015	*Chenopodiaceae*	*Chenopodium berlandieri*	+	32º20′06.3″ N, 114º53′31.4″ W
SO-HU	2015	*Chenopodiaceae*	*Chenopodium spp*	+	26º 24′54.5″N, 109º01′15.1″W
SO-HU	2015	*Chenopodiaceae*	*Chenopodium spp*		26º 24′54.5″N, 109º01′15.1″W
SO-HU	2015	*Convolvulaceae*	*Convolvulus arvensis*	+	26º 24′54.5″N, 109º01′15.1″W
SO-OB	2015	*Convolvulaceae*	*Convolvulus arvensis*	+	27º 08′09.9″N, 109º53′31.7″W
SO-HU	2015	*Euphorbiaceae*	*Cnidoscolus spp*		26º50'05.9"N, 109º31'51.8"

SO-HU	2015	*Euphorbiaceae*	*Cnidoscolus spp*	+	26º50'05.9"N, 109º31'51.8"
SO-OB	2015	*Euphorbiaceae*	*Ricinus communis*	+	27º 08'09.9"N, 109º53'31.7"W
SO-NA	2015	*Fabaceae*	*Meliotus indica*		26º99'98.46"N, 109º52'36.35" W
SO-NA	2015	*Fabaceae*	*Meliotus indica*	+	26º96'00.39''N, 109º58'24.36'' W
SO-OB	2015	*Hydrophyllaceae*	*Nama jamaicensis*	+	27º 36'22.5"N, 110º08'02.5"W
SO-HU	2015	*Malvaceae*	*Abutilon palmeri*	+	26º 24'54.5"N, 109º01'15.1"W
SO-HU	2015	*Malvaceae*	*Abutilon palmeri*	+	26º 24'54.5"N, 109º01'15.1"W
SO-OB	2015	*Malvaceae*	*Abutilon palmeri*	+	27º 36'22.5"N, 110º08'02.5"W
SO-HU	2015	*Malvaceae*	*Abutilon trisulcatun*	+	26º 24'54.5"N, 109º01'15.1"W
SO-HU	2015	*Malvaceae*	*Anoda pedunculosa*	+	26º 24'54.5"N, 109º01'15.1"W
SO-HU	2015	*Malvaceae*	*Malva parviflora*		26º 24'54.5"N, 109º01'15.1"W
SO-NA	2015	*Malvaceae*	*Malva parviflora*	+	26º90'05.02''N, 109º52'28.42'' W
SO-NA	2015	*Malvaceae*	*Malva parviflora*	+	26º90'05.02''N, 109º52'28.42'' W
SO-NA	2015	*Malvaceae*	*Malva parviflora*	+	26º90'05.02''N, 109º52'28.42'' W
SO-NA	2015	*Malvaceae*	*Malva parviflora*	+	26º 86' 23.43''N, 109º68'63.83 W
SO-NA	2015	*Malvaceae*	*Malva parviflora*	+	26º 86' 23.43''N, 109º68'63.83 W
SO-NA	2015	*Malvaceae*	*Malva parviflora*	+	26º96'00.39''N, 109º58'24.36'' W
SO-RC	2015	*Malvaceae*	*Malva parviflora*	+	32º25'07.8" N, 114º49'34.7" W
SO-NA	2015	*Malvaceae*	*Malvella leprosa*	+	26º90'05.02''N, 109º52'28.42'' W
SO-NA	2015	*Malvaceae*	*Malvella leprosa*		26º90'05.02''N, 109º52'28.42'' W
SO-NA	2015	*Malvaceae*	*Malvella leprosa*		26º90'05.02''N, 109º52'28.42'' W
SO-OB	2015	*Malvaceae*	*Malvella leprosa*		27º 08'09.9"N, 109º53'31.7"W
SO-OB	2015	*Malvaceae*	*Sida rombifolia*	+	27º 36'22.5"N, 110º08'02.5"W
SO-HU	2015	*Onagraceae*	*Ludwigia octovalvis*		26º50'05.9"N, 109º31'51.8"
SO-HU	2015	*Onagraceae*	*Ludwigia octovalvis*	+	26º50'05.9"N, 109º31'51.8"
SO-OB	2015	*Papaveraceae*	*Argemone mexicana*	+	27º 36'22.5"N, 110º08'02.5"W
SO-HU	2015	*Polygonaceae*	*Antigonon leptopus*	+	26º 24'54.5"N, 109º01'15.1"W
SO-NA	2015	*Polygonaceae*	*Rumex crispo*		26º90'05.02''N, 109º52'28.42'' W
SO-NA	2015	*Polygonaceae*	*Rumex crispo*	+	26º90'05.02''N, 109º52'28.42'' W
SO-NA	2015	*Polygonaceae*	*Rumex crispo*	+	26º90'05.02''N, 109º52'28.42'' W
SO-OB	2015	*Polygonaceae*	*Rumex crispo*		27º 08'09.9"N, 109º53'31.7"W
SO-HU	2015	*Rhamnaceae*	*Gouania lupuloides*	+	26º 24'54.5"N, 109º01'15.1"W
SO-HU	2015	*Solanaceae*	*Datura stramonium*	+	26º47'06.1"N, 109º40'52.8"W
SO-HU	2015	*Solanaceae*	*Nicotiana glauca*	+	26º50'05.9"N, 109º31'51.8"
SO-HU	2015	*Solanaceae*	*Nicotiana glauca*		26º50'05.9"N, 109º31'51.8"
SO-HU	2015	*Solanaceae*	*Nicotiana glauca*		26º50'05.9"N, 109º31'51.8"
SO-HU	2015	*Solanaceae*	*Nicotiana glauca*	+	26º47'06.1"N, 109º40'52.8"W
SO-OB	2015	*Solanaceae*	*Nicotiana glauca*	+	27º 08'09.9"N, 109º53'31.7"W
SO-HU	2015	*Solanaceae*	*Nicotiana plumbanginifolia*	+	26º50'05.9"N, 109º31'51.8"
SO-HU	2015	*Solanaceae*	*Nicotina plumbanginifolia*	+	26º 24'54.5"N, 109º01'15.1"W

SO-OB	2015	*Solanaceae*	*Nicotina plumbanginifolia*	+	27º 36’22.5”N, 110º08’02.5”W
SO-HU	2015	*Solanaceae*	*Solanum lycopersicum*	+	26º47’06.1”N, 109º40’52.8”W
SO-HU	2015	*Solanaceae*	*Solanum nigrescens*	+	26º 24’54.5”N, 109º01’15.1”W
SO-HU	2015	*Solanaceae*	*Solanum pseudocapsicum*		26º 24’54.5”N, 109º01’15.1”W
SO-HU	2015	*Solanaceae*	*Solanum spp*	+	26º47’06.1”N, 109º40’52.8”W
SO-HU	2015	*Solanaceae*	*Solanum verbascifolium*	+	26º50'05.9"N, 109º31'51.8"
SI-CO	2012	*Amaranthaceae*	*Amaranthus palmeri*		23º17'13.489"N, 106º4'51.193" W
SI-GV	2012	*Amaranthaceae*	*Amaranthus palmeri*		25º44'59.049" N, 108º39'46.189 W
SI-GV	2012	*Amaranthaceae*	*Amaranthus palmeri*	+	25º43'49.164" N, 108º24'27.845" W
SI-SL	2012	*Amaranthaceae*	*Amaranthus palmeri*		25º49'29.732"N, 108º14'4.196" W
SI-SL	2012	*Apocynaceae*	*Stemmadenia palmeri*		25º48'42.44" N, 108º13'9.302" W
SI-CO	2012	*Asclepiadiaceae*	*Sarcostema spp*		23º17'13.489"N, 106º4'51.193" W
SI-SL	2012	*Asteraceae*	*Artemisia ludoviciana*		25º48'42.44" N, 108º13'9.302" W
SI-SL	2012	*Asteraceae*	*Franseria ambrosioides*		25º48'42.44" N, 108º13'9.302" W
SI-GV	2012	*Asteraceae*	*Helianthus annuus*		25º43'49.164" N, 108º24'27.845" W
SI-GV	2014	*Asteraceae*	*Parthenium hysterophorus*		20º30'39.585" N, 108º30'39.252" W
SI-GV	2014	*Asteraceae*	*Parthenium hysterophorus*		20º30'39.585" N, 108º30'39.252" W
SI-GV	2014	*Asteraceae*	*Parthenium hysterophorus*		20º30'39.585" N, 108º30'39.252" W
SI-GV	2014	*Asteraceae*	*Parthenium hysterophorus*		20º30'39.585" N, 108º30'39.252" W
SI-GV	2012	*Asteraceae*	*Parthenium hysterophorus*		25º43'49.164" N, 108º24'27.845" W
SI-GV	2012	*Asteraceae*	*Parthenium hysterophorus*	+	25º43'49.164" N, 108º24'27.845" W
SI-GV	2012	*Asteraceae*	*Parthenium hysterophorus*		25º43'49.164" N, 108º24'27.845" W
SI-GV	2012	*Asteraceae*	*Parthenium hysterophorus*	+	25º43'49.164" N, 108º24'27.845" W
SI-GV	2012	*Asteraceae*	*Parthenium hysterophorus*		25º43'49.164" N, 108º24'27.845" W
SI-GV	2012	*Asteraceae*	*Parthenium hysterophorus*	+	25º43'49.164" N, 108º24'27.845" W
SI-GV	2012	*Asteraceae*	*Parthenium hysterophorus*	+	25º43'49.164" N, 108º24'27.845" W
SI-MO	2012	*Asteraceae*	*Parthenium hysterophorus*		25º28'55.277" N, 107º54'14.071" W
SI-RO	2012	*Asteraceae*	*Parthenium hysterophorus*	+	23º0'8.665"N, 105º51'26.246" W
SI-RO	2012	*Asteraceae*	*Parthenium hysterophorus*		23º0'8.665"N, 105º51'26.246" W
SI-GV	2012	*Asteraceae*	*Porophyllum punctatum*	+	25º43'49.164" N, 108º24'27.845" W
SI-GV	2012	*Asteraceae*	*Sonchus oleraceus*	+	25º43'49.164" N, 108º24'27.845" W
SI-GV	2012	*Asteraceae*	*Sonchus oleraceus*		25º43'49.164" N, 108º24'27.845" W
SI-SL	2012	*Asteraceae*	*Sonchus oleraceus*		25º48'42.44" N, 108º13'9.302" W
SI-GV	2012	*Asteraceae*	*Xanthium strumarium*	+	25º43'49.164" N, 108º24'27.845" W
SI-MO	2012	*Bignoniaceae*	*Amphilophium paniculatum*		25º28'55.277" N, 107º54'14.071" W
SI-GV	2015	*Brassicaceae*	*Diplotaxis muralis*	+	32º25’07.8” N, 114º49’34.7” W
SI-SL	2012	*Caesalpiniaceae*	*Caesalpinia platyloba*	+	25º48'42.44" N, 108º13'9.302" W
SI-CO	2012	*Capparaceae*	*Polinesia dodecandra*	+	23º17'13.489"N, 106º4'51.193" W
SI-SL	2012	*Capparaceae*	*Polinesia dodecandra*		25º49'29.732"N, 108º14'4.196" W
SI-GV	2012	*Convolvulaceae*	*Ipomoea purpurea*		25º43'49.164" N, 108º24'27.845" W

SI-CO	2012	*Convolvulaceae*	*Ipomoea spp*	+	23º17'13.489"N, 106º4'51.193" W
SI-GV	2012	*Convolvulaceae*	*Ipomoea spp*	+	25º44'59.049" N, 108º39'46.189 W
SI-GV	2012	*Convolvulaceae*	*Ipomoea spp*	+	25º44'59.049" N, 108º39'46.189 W
SI-SL	2012	*Cucurbitaceae*	*Citrullus lanatus*		25º48'42.44" N, 108º13'9.302" W
SI-GV	2012	*Cucurbitaceae*	*Cucumis anguria*		25º44'59.049" N, 108º39'46.189 W
SI-GV	2012	*Cucurbitaceae*	*Cucumis dipsaceus*	+	25º43'49.164" N, 108º24'27.845" W
SI-GV	2012	*Cucurbitaceae*	*Momardica charantia*	+	25º43'49.164" N, 108º24'27.845" W
SI-MO	2012	*Cucurbitaceae*	*Momardica charantia*		25º28'55.277" N, 107º54'14.071" W
SI-MO	2012	*Cucurbitaceae*	*Momardica charantia*		25º28'55.277" N, 107º54'14.071" W
SI-CO	2012	*Euphorbiaceae*	*Acalypha polystachya*		23º17'13.489"N, 106º4'51.193" W
SI-GV	2012	*Euphorbiaceae*	*Acalypha polystachya*	+	25º43'49.164" N, 108º24'27.845" W
SI-MO	2012	*Euphorbiaceae*	*Acalypha polystachya*		25º28'55.277" N, 107º54'14.071" W
SI-MO	2012	*Euphorbiaceae*	*Acalypha polystachya*		25º28'55.277" N, 107º54'14.071" W
SI-MO	2012	*Euphorbiaceae*	*Croton spp*		25º28'55.277" N, 107º54'14.071" W
SI-MO	2012	*Euphorbiaceae*	*Euphorbia heterophylla*		25º28'55.277" N, 107º54'14.071" W
SI-RO	2014	*Euphorbiaceae*	*Euphorbia heterophylla*	+	23º0'8.665"N, 105º51'26.246" W
SI-RO	2014	*Euphorbiaceae*	*Euphorbia heterophylla*	+	23º0'8.665"N, 105º51'26.246" W
SI-RO	2014	*Euphorbiaceae*	*Euphorbia heterophylla*	+	23º0'8.665"N, 105º51'26.246" W
SI-RO	2015	*Euphorbiaceae*	*Euphorbia heterophylla*		23º0'8.665"N, 105º51'26.246" W
SI-RO	2015	*Euphorbiaceae*	*Euphorbia heterophylla*		23º0'8.665"N, 105º51'26.246" W
SI-MO	2012	*Euphorbiaceae*	*Manihot spp*	+	25º28'55.277" N, 107º54'14.071" W
SI-GV	2014	*Euphorbiaceae*	*Ricinus communis*		20º30'39.585" N, 108º30'39.252" W
SI-GV	2012	*Euphorbiaceae*	*Ricinus communis*		25º43'49.164" N, 108º24'27.845" W
SI-SL	2012	*Euphorbiaceae*	*Ricinus communis*		25º48'42.44" N, 108º13'9.302" W
SI-GV	2016	*Fabaceae*	*Crotalaria juncea*	+	25º43'01.45" N, 108º19'42.45 W
SI-GV	2016	*Fabaceae*	*Crotalaria juncea*	+	25º43'01.45" N, 108º19'42.45 W
SI-GV	2016	*Fabaceae*	*Crotalaria juncea*	+	25º43'01.45" N, 108º19'42.45 W
SI-GV	2012	*Fabaceae*	*Lonchocarpus lanceolatus*	+	25º43'01.45" N, 108º19'42.45 W
SI-ES	2016	*Fabaceae*	*Macroptilium atropurpureum*		23º00'08.66 N, 105º51'26.23 W
SI-ES	2016	*Fabaceae*	*Macroptilium atropurpureum*		23º00'08.66 N, 105º51'26.23 W
SI-ES	2016	*Fabaceae*	*Macroptilium atropurpureum*		23º00'08.66 N, 105º51'26.23 W
SI-RO	2016	*Fabaceae*	*Macroptilium atropurpureum*	+	23º00'08.66 N, 105º51'26.23 W
SI-RO	2016	*Fabaceae*	*Macroptilium atropurpureum*	+	23º00'08.66 N, 105º51'26.23 W
SI-RO	2016	*Fabaceae*	*Macroptilium atropurpureum*	+	23º00'08.66 N, 105º51'26.23 W
SI-RO	2015	*Fabaceae*	*Macroptilium atropurpureum*		23º00'08.66 N, 105º51'26.23 W
SI-RO	2016	*Fabaceae*	*Macroptilium atropurpureum*	+	23º00'08.66 N, 105º51'26.23 W
SI-GV	2015	*Fabaceae*	*Melilotus indicus*	+	23º00'08.66 N, 105º51'26.23 W
SI-GV	2016	*Fabaceae*	*Melilotus indicus*	+	23º00'08.66 N, 105º51'26.23 W
SI-AB	2016	*Fabaceae*	*Rhynchosia minima*		22º34'27.113" N, 108º32'30.951" W
SI-AB	2016	*Fabaceae*	*Rhynchosia minima*		22º34'27.113" N, 108º32'30.951" W

SI-AB	2016	*Fabaceae*	*Rhynchosia minima*	+	22º34'27.113" N, 108º32'30.951" W
SI-AB	2016	*Fabaceae*	*Rhynchosia minima*	+	22º34'27.113" N, 108º32'30.951" W
SI-AB	2016	*Fabaceae*	*Rhynchosia minima*	+	22º34'27.113" N, 108º32'30.951" W
SI-AC	2016	*Fabaceae*	*Rhynchosia minima*	+	23º09'39.09" N, 106º05'26.09" W
SI-GV	2012	*Fabaceae*	*Rhynchosia minima*	+	25º44'59.049" N, 108º39'46.188" W
SI-GV	2016	*Fabaceae*	*Rhynchosia minima*		25º32'31.16" N, 108º32'46.154" W
SI-GV	2016	*Fabaceae*	*Rhynchosia minima*		25º32'31.16" N, 108º32'46.154" W
SI-GV	2016	*Fabaceae*	*Rhynchosia minima*	+	25º32'31.16" N, 108º32'46.154" W
SI-GV	2016	*Fabaceae*	*Rhynchosia minima*	+	25º32'31.16" N, 108º32'46.154" W
SI-GV	2016	*Fabaceae*	*Rhynchosia minima*	+	25º32'31.16" N, 108º32'46.154" W
SI-GV	2014	*Fabaceae*	*Rhynchosia minima*		25º32'31.16" N, 108º32'46.154" W
SI-GV	2014	*Fabaceae*	*Rhynchosia minima*	+	25º32'31.16" N, 108º32'46.154" W
SI-GV	2014	*Fabaceae*	*Rhynchosia minima*	+	25º32'31.16" N, 108º32'46.154" W
SI-GV	2014	*Fabaceae*	*Rhynchosia minima*	+	25º32'31.16" N, 108º32'46.154" W
SI-PC	2016	*Fabaceae*	*Rhynchosia minima*	+	23º50'37.66" N, 107º00'33.67" W
SI-PC	2016	*Fabaceae*	*Rhynchosia minima*	+	23º50'37.66" N, 107º00'33.67" W
SI-PC	2016	*Fabaceae*	*Rhynchosia minima*		23º50'37.66" N, 107º00'33.67" W
SI-PC	2016	*Fabaceae*	*Rhynchosia minima*	+	23º50'37.66" N, 107º00'33.67" W
SI-PC	2016	*Fabaceae*	*Rhynchosia minima*	+	23º50'37.66" N, 107º00'33.67" W
SI-PC	2016	*Fabaceae*	*Rhynchosia minima*	+	23º50'37.66" N, 107º00'33.67" W
SI-RO	2016	*Fabaceae*	*Rhynchosia minima*		23º0'8.665"N, 105º51'26.246" W
SI-RO	2014	*Fabaceae*	*Rhynchosia minima*	+	23º00'08.66 N, 105º51'26.24 W
SI-RO	2014	*Fabaceae*	*Rhynchosia minima*		23º00'08.66 N, 105º51'26.24 W
SI-AC	2016	*Fabaceae*	*Rhynchosia precatoria*	+	23º09'39.09" N, 106º05'26.09" W
SI-AC	2016	*Fabaceae*	*Rhynchosia precatoria*		23º09'39.09" N, 106º05'26.09" W
SI-AC	2016	*Fabaceae*	*Rhynchosia precatoria*	+	23º09'39.09" N, 106º05'26.09" W
SI-AC	2016	*Fabaceae*	*Rhynchosia precatoria*	+	23º09'39.09" N, 106º05'26.09" W
SI-AC	2016	*Fabaceae*	*Rhynchosia precatoria*		23º09'39.09" N, 106º05'26.09" W
SI-AC	2016	*Fabaceae*	*Rhynchosia precatoria*	+	23º09'39.09" N, 106º05'26.09" W
SI-AC	2016	*Fabaceae*	*Rhynchosia precatoria*		23º09'39.09" N, 106º05'26.09" W
SI-ES	2016	*Fabaceae*	*Rhynchosia precatoria*	+	22º58'7.266"N, 105º54'34.657"W
SI-ES	2016	*Fabaceae*	*Rhynchosia precatoria*		22º58'7.266"N, 105º54'34.657"W
SI-ES	2016	*Fabaceae*	*Rhynchosia precatoria*	+	22º58'7.266"N, 105º54'34.657"W
SI-ES	2016	*Fabaceae*	*Rhynchosia precatoria*		22º58'7.266"N, 105º54'34.657"W
SI-ES	2016	*Fabaceae*	*Rhynchosia precatoria*		22º58'7.266"N, 105º54'34.657"W
SI-RO	2016	*Fabaceae*	*Rhynchosia precatoria*	+	23º00'08.66 N, 105º51'26.24 W
SI-RO	2016	*Fabaceae*	*Rhynchosia precatoria*		23º00'08.66 N, 105º51'26.24 W
SI-RO	2016	*Fabaceae*	*Rhynchosia precatoria*	+	23º00'08.66 N, 105º51'26.24 W
SI-RO	2016	*Fabaceae*	*Rhynchosia precatoria*	+	23º00'08.66 N, 105º51'26.24 W
SI-RO	2014	*Fabaceae*	*Rhynchosia precatoria*		23º00'08.66 N, 105º51'26.24 W

SI-RO	2016	*Fabaceae*	*Rhynchosia precatoria*		23º00'08.66 N, 105º51'26.24 W
SI-AC	2016	*Fabaceae*	*Rhynchosia minima*	+	23º09'39.09" N, 106º05'26.09" W
SI-AC	2016	*Fabaceae*	*Rhynchosia minima*	+	23º09'39.09" N, 106º05'26.09" W
SI-AC	2016	*Fabaceae*	*Rhynchosia minima*	+	23º09'39.09" N, 106º05'26.09" W
SI-AC	2016	*Fabaceae*	*Rhynchosia minima*	+	23º09'39.09" N, 106º05'26.09" W
SI-AC	2016	*Fabaceae*	*Rhynchosia minima*	+	23º09'39.09" N, 106º05'26.09" W
SI-PC	2016	*Fabaceae*	*Rhynchosia minima*	+	23º50'37.66" N, 107º00'33.67" W
SI-PC	2016	*Fabaceae*	*Rhynchosia minima*	+	23º50'37.66" N, 107º00'33.67" W
SI-RO	2016	*Fabaceae*	*Rhynchosia minima*	+	23º00'08.66 N, 105º51'26.24 W
SI-RO	2016	*Fabaceae*	*Rhynchosia minima*	+	23º00'08.66 N, 105º51'26.24 W
SI-RO	2016	*Fabaceae*	*Rhynchosia minima*	+	23º00'08.66 N, 105º51'26.24 W
SI-RO	2016	*Fabaceae*	*Rhynchosia minima*	+	23º00'08.66 N, 105º51'26.24 W
SI-RO	2016	*Fabaceae*	*Senna uniflora*	+	23º00'08.66 N, 105º51'26.24 W
SI-RO	2014	*Fabaceae*	*Senna uniflora*	+	23º00'08.66 N, 105º51'26.24 W
SI-RO	2015	*Fabaceae*	*Senna uniflora*		23º00'08.66 N, 105º51'26.24 W
SI-SL	2012	*Fabaceae*	*Senna uniflora*		25º48'42.44" N, 108º13'9.302" W
SI-SL	2012	*Malpiguiaceae*	*Mascagnia macroptera*		25º48'42.44" N, 108º13'9.302" W
SI-SL	2012	*Malpiguiaceae*	*Mascagnia macroptera*		25º48'42.44" N, 108º13'9.302" W
SI-GV	2012	*Malvaceae*	*Abutilon palmeri*	+	25º43'49.164" N, 108º24'27.845" W
SI-GV	2012	*Malvaceae*	*Abutilon palmeri*		25º43'49.164" N, 108º24'27.845" W
SI-SL	2012	*Malvaceae*	*Abutilon palmeri*		25º48'42.44" N, 108º13'9.302" W
SI-GV	2014	*Malvaceae*	*Abutilon trisulcatun*	+	20º30'39.585" N, 108º30'39.252" W
SI-GV	2014	*Malvaceae*	*Abutilon trisulcatun*		20º30'39.585" N, 108º30'39.252" W
SI-GV	2014	*Malvaceae*	*Abutilon trisulcatun*	+	20º30'39.585" N, 108º30'39.252" W
SI-GV	2014	*Malvaceae*	*Abutilon trisulcatun*	+	20º30'39.585" N, 108º30'39.252" W
SI-GV	2012	*Malvaceae*	*Abutilon trisulcatun*	+	25º43'49.164" N, 108º24'27.845" W
SI-MO	2012	*Malvaceae*	*Abutilon trisulcatun*	+	25º28'55.277" N, 107º54'14.071" W
SI-MO	2012	*Malvaceae*	*Abutilon trisulcatun*	+	25º28'55.277" N, 107º54'14.071" W
SI-MO	2012	*Malvaceae*	*Abutilon trisulcatun*		25º28'55.277" N, 107º54'14.071" W
SI-MO	2012	*Malvaceae*	*Abutilon trisulcatun*		25º28'55.277" N, 107º54'14.071" W
SI-MO	2012	*Malvaceae*	*Abutilon trisulcatun*		25º28'55.277" N, 107º54'14.071" W
SI-RO	2014	*Malvaceae*	*Abutilon trisulcatun*	+	23º0'8.665"N, 105º51'26.246" W
SI-RO	2015	*Malvaceae*	*Abutilon trisulcatun*		23º0'8.665"N, 105º51'26.246" W
SI-CO	2012	*Malvaceae*	*Anoda pentaschista*	+	23º17'13.489"N, 106º4'51.193" W
SI-GV	2012	*Malvaceae*	*Herisantia crispa*	+	25º43'49.164" N, 108º24'27.845" W
SI-RO	2014	*Malvaceae*	*Herisantia crispa*	+	23º0'8.665"N, 105º51'26.246" W
SI-GV	2012	*Malvaceae*	*Kosteletzkya depressa*	+	25º43'49.164" N, 108º24'27.845" W
SI-GV	2012	*Malvaceae*	*Kosteletzkya depressa*	+	25º43'49.164" N, 108º24'27.845" W
SI-GV	2014	*Malvaceae*	*Melochia piramydata*	+	20º30'39.585" N, 108º30'39.252" W
SI-GV	2014	*Malvaceae*	*Melochia piramydata*	+	20º30'39.585" N, 108º30'39.252" W

SI-GV	2014	*Malvaceae*	*Melochia piramydata*	+	20º30'39.585" N, 108º30'39.252" W
SI-GV	2014	*Malvaceae*	*Melochia piramydata*	+	20º30'39.585" N, 108º30'39.252" W
SI-RO	2014	*Malvaceae*	*Melochia piramydata*	+	23º0'8.665"N, 105º51'26.246" W
SI-GV	2014	*Malvaceae*	*Sida acuta*		20º30'39.585" N, 108º30'39.252" W
SI-GV	2014	*Malvaceae*	*Sida acuta*		20º30'39.585" N, 108º30'39.252" W
SI-RO	2014	*Malvaceae*	*Sida acuta*	+	23º0'8.665"N, 105º51'26.246" W
SI-RO	2014	*Malvaceae*	*Sida acuta*	+	23º0'8.665"N, 105º51'26.246" W
SI-RO	2015	*Malvaceae*	*Sida acuta*		23º0'8.665"N, 105º51'26.246" W
SI-MO	2012	*Malvaceae*	*Sidastrum lodiegensis*	+	25º28'55.277" N, 107º54'14.071" W
SI-MO	2012	*Malvaceae*	*Sidastrum lodiegensis*		25º28'55.277" N, 107º54'14.071" W
SI-MO	2012	*Malvaceae*	*Sidastrum lodiegensis*		25º28'55.277" N, 107º54'14.071" W
SI-GV	2012	*Menispermaceae*	*Coccolus diversifolius*	+	25º43'49.164" N, 108º24'27.845" W
SI-MO	2012	*Nyctaginaceae*	*Boerhavia spp*	+	25º28'55.277" N, 107º54'14.071" W
SI-GV	2012	*Nyctaginaceae*	*Salpianthus macrodonthus*	+	25º43'49.164" N, 108º24'27.845" W
SI-GV	2012	*Onagraceae*	*Ludwigia erecta*		25º44'59.049" N, 108º39'46.189 W
SI-GV	2012	*Onagraceae*	*Ludwigia octovalvis*		25º44'59.049" N, 108º39'46.189 W
SI-CO	2012	*Pedaliaceae*	*Matynia annua*	+	23º17'13.489"N, 106º4'51.193" W
SI-GV	2012	*Portulacaceae*	*Portulaca oleraceae*	+	25º44'59.049" N, 108º39'46.189 W
SI-GV	2012	*Portulacaceae*	*Portulaca oleraceae*	+	25º48'42.44" N, 108º13'9.302" W
SI-GV	2012	*Portulacaceae*	*Portulaca oleraceae*	+	25º48'42.44" N, 108º13'9.302" W
SI-SL	2012	*Rhamnaceae*	*Karwinskia humboldtiana*	+	25º48'42.44" N, 108º13'9.302" W
SI-CO	2012	*Rubiaceae*	*Borreria laevis*		23º17'13.489"N, 106º4'51.193" W
SI-GV	2012	*Sapindaceae*	*Paullinia fuscescens*	+	25º43'49.164" N, 108º24'27.845" W
SI-MO	2012	*Solanaceae*	*Datura discolor*	+	25º28'55.277" N, 107º54'14.071" W
SI-MO	2012	*Solanaceae*	*Datura discolor*	+	25º28'55.277" N, 107º54'14.071" W
SI-SL	2012	*Solanaceae*	*Datura inoxia*	+	25º48'42.44" N, 108º13'9.302" W
SI-GV	2012	*Solanaceae*	*Datura reburra*	+	25º43'49.164" N, 108º24'27.845" W
SI-GV	2012	*Solanaceae*	*Datura stramonium*	+	25º44'59.049" N, 108º39'46.189 W
SI-GV	2014	*Solanaceae*	*Datura stramonium*	+	20º30'39.585" N, 108º30'39.252" W
SI-GV	2014	*Solanaceae*	*Datura stramonium*	+	20º30'39.585" N, 108º30'39.252" W
SI-GV	2014	*Solanaceae*	*Datura stramonium*	+	20º30'39.585" N, 108º30'39.252" W
SI-GV	2014	*Solanaceae*	*Datura stramonium*	+	20º30'39.585" N, 108º30'39.252" W
SI-GV	2012	*Solanaceae*	*Nicotiana glauca*	+	25º44'59.049" N, 108º39'46.189 W
SI-GV	2012	*Solanaceae*	*Nicotiana glauca*	+	25º43'49.164" N, 108º24'27.845" W
SI-RO	2014	*Solanaceae*	*Physalis acutifolia*	+	23º0'8.665"N, 105º51'26.246" W
SI-MO	2012	*Solanaceae*	*Physalis Angulata*		25º28'55.277" N, 107º54'14.071" W
SI-GV	2012	*Solanaceae*	*Solanum americanum*	+	25º43'49.164" N, 108º24'27.845" W
SI-CO	2012	*Solanaceae*	*Solanum nigrescens*		23º17'13.489"N, 106º4'51.193" W
SI-GV	2012	*Solanaceae*	*Solanum nigrescens*	+	25º43'49.164" N, 108º24'27.845" W
SI-MO	2012	*Solanaceae*	*Solanum nigrescens*		25º28'55.277" N, 107º54'14.071" W

SI-MO	2012	*Solanaceae*	*Solanum tridynamum*	+	25º28'55.277" N, 107º54'14.071" W
SI-MO	2012	*Solanaceae*	*Solanum tridynamum*		25º28'55.277" N, 107º54'14.071" W
SI-SL	2012	*Solanaceae*	*Solanum tridynamum*	+	25º48'42.44" N, 108º13'9.302" W
SI-SL	2012	*Solanaceae*	*Solanum tridynamum*	+	25º48'42.44" N, 108º13'9.302" W
SI-SL	2012	*Solanaceae*	*Solanum tridynamum*	+	25º48'42.44" N, 108º13'9.302" W
SI-CO	2012	*Sterculiaceae*	*Melochia piramydata*		23º17'13.489"N, 106º4'51.193" W
SI-CO	2012	*Sterculiaceae*	*Waltheria americana*	+	23º17'13.489"N, 106º4'51.193" W
SI-GV	2012	*Verbenaceae*	*Verbenaceae spp*	+	25º43'49.164" N, 108º24'27.845" W
SI-GV	2012	*Verbenaceae*	*Verbenaceae spp*		25º43'49.164" N, 108º24'27.845" W
SI-MO	2012	*Vitaceae*	*Vitaceae spp*	+	25º28'55.277" N, 107º54'14.071" W
CN-SM	2014	*Amaranthaceae*	*Amaranthus hybridus*	+	21º20'35.76" N, 104º40'15.124" W
CN-SM	2014	*Amaranthaceae*	*Amaranthus retroflexus*		21º20'35.76" N, 104º40'15.124" W
CN-TE	2014	*Amaranthaceae*	*Amaranthus spinosus*		18º50'53.07" N, 103º49'54.892" W
CN-TE	2014	*Amaranthaceae*	*Amaranthus spinosus*		18º50'53.07" N, 103º49'54.892" W
CN-SM	2014	*Asteraceae*	*Cosmos sulphureus*	+	21º20'35.76" N, 104º40'15.124" W
CN-SM	2014	*Asteraceae*	*Cosmos sulphureus*	+	21º20'35.76" N, 104º40'15.124" W
CN-TE	2014	*Asteraceae*	*Erigeron longipes*		18º50'53.07" N, 103º49'54.892" W
CN-SM	2014	*Asteraceae*	*Melampodium rosei*	+	21º20'35.76" N, 104º40'15.124" W
CN-TE	2014	*Commelinaceae*	*Commelina diffusa*		18º50'53.07" N, 103º49'54.892" W
CN-SM	2014	*Convolvulaceae*	*Ipomoea hederaceae*		21º20'35.76" N, 104º40'15.124" W
CN-SM	2014	*Convolvulaceae*	*Ipomoea spp.*		21º20'35.76" N, 104º40'15.124" W
CN-SM	2014	*Convolvulaceae*	*Ipomoea spp.*	+	21º20'35.76" N, 104º40'15.124" W
CN-TE	2014	*Convolvulaceae*	*Merremia quinquefolia*		18º50'53.07" N, 103º49'54.892" W
CN-TE	2014	*Cucurbitaceae*	*Momardica charantia*		18º50'53.07" N, 103º49'54.892" W
CN-TE	2014	*Cucurbitaceae*	*Momardica charantia*		18º50'53.07" N, 103º49'54.892" W
CN-TE	2014	*Cucurbitaceae*	*Momardica charantia*	+	18º50'53.07" N, 103º49'54.892" W
CN-TE	2014	*Euphorbiaceae*	*Acalypha seetosa*		18º50'53.07" N, 103º49'54.892" W
CN-TE	2014	*Euphorbiaceae*	*Euphorbia hypericifolia*		18º50'53.07" N, 103º49'54.892" W
CN-TE	2014	*Fabaceae*	*Rhynchosia spp*		18º50'53.07" N, 103º49'54.892" W
CN-SM	2014	*Hydrophyllaceae*	*Nama hispida*		21º20'35.76" N, 104º40'15.124" W
CN-TE	2014	*Malvaceae*	*Herisantia crispa*		18º50'53.07" N, 103º49'54.892" W
CN-TE	2014	*Malvaceae*	*Herisantia crispa*	+	18º50'53.07" N, 103º49'54.892" W
CN-TE	2014	*Malvaceae*	*Herisantia crispa*		18º50'53.07" N, 103º49'54.892" W
CN-TE	2014	*Malvaceae*	*Herisantia crispa*	+	18º50'53.07" N, 103º49'54.892" W
CN-TE	2014	*Malvaceae*	*Herisantia crispa*	+	18º50'53.07" N, 103º49'54.892" W
CN-TE	2014	*Malvaceae*	*Herisantia crispa*	+	18º50'53.07" N, 103º49'54.892" W
CN-TE	2014	*Malvaceae*	*Herisantia crispa*	+	18º50'53.07" N, 103º49'54.892" W
CN-TE	2014	*Malvaceae*	*Herisantia crispa*		18º50'53.07" N, 103º49'54.892" W
CN-TE	2014	*Malvaceae*	*Malvastrum coromandelianum*	+	18º50'53.07" N, 103º49'54.892" W
CN-TE	2014	*Malvaceae*	*Malvastrum coromandelianum*	+	18º50'53.07" N, 103º49'54.892" W

CN-TE	2014	*Malvaceae*	*Malvastrum coromandelianum*	+	18º50'53.07" N, 103º49'54.892" W
CN-TE	2014	*Malvaceae*	*Sida acuta*	+	18º50'53.07" N, 103º49'54.892" W
CN-SM	2014	*Malvaceae*	*Sida collina*		21º20'35.76" N, 104º40'15.124" W
CN-TE	2014	*Nyctaginaceae*	*Boerhavia coccinea*		18º50'53.07" N, 103º49'54.892" W
CN-TE	2014	*Nyctaginaceae*	*Boerhavia coccinea*	+	18º50'53.07" N, 103º49'54.892" W
CN-TE	2014	*Nyctaginaceae*	*Boerhavia coccinea*	+	18º50'53.07" N, 103º49'54.892" W
CN-TE	2014	*Nyctaginaceae*	*Boerhavia coccinea*	+	18º50'53.07" N, 103º49'54.892" W
CN-TE	2014	*Nyctaginaceae*	*Boerhavia coccinea*	+	18º50'53.07" N, 103º49'54.892" W
CN-SM	2014	*Portulacaceae*	*Portulaca oleraceae*		21º20'35.76" N, 104º40'15.124" W
CN-TE	2014	*Portulcaaceae*	*Portulaca oleraceae*	+	18º50'53.07" N, 103º49'54.892" W
CN-TE	2014	*Rubiaceae*	*Galium mexicanum*	+	18º50'53.07" N, 103º49'54.892" W
CN-TE	2014	*Rubiaceae*	*Richardia scabia*	+	18º50'53.07" N, 103º49'54.892" W
CN-TE	2014	*Solanaceae*	*Solanum nigrescens*		18º50'53.07" N, 103º49'54.892" W
CN-TE	2014	*Solanaceae*	*Solanum nigrescens*		18º50'53.07" N, 103º49'54.892" W
CN-TE	2014	*Verbenaceae*	*Priva iappulaceae*	+	18º50'53.07" N, 103º49'54.892" W
CN-SM	2014	*Verbenaceae*	*Verbenaceae spp*	+	21º20'35.76" N, 104º40'15.124" W
CN-TE	2014	*Vitaceae*	*Cissus sicyoides*		18º50'53.07" N, 103º49'54.892" W
CD-LG	2015	*Amaranthaceae*	*Amaranthus spp*	+	25º29'28.04'' N, 103º41'12.80 W
CD-LG	2015	*Amaranthaceae*	*Amaranthus spp*	+	25º29'28.04'' N, 103º41'12.80 W
CD-PO	2016	*Amaranthaceae*	*Amaranthus spp*	+	24º00'37.45'' N, 104º07'31.73 W
CD-PO	2016	*Amaranthaceae*	*Amaranthus spp*	+	24º00'37.45'' N, 104º07'31.73 W
CD-PO	2016	*Amaranthaceae*	*Amaranthus spp*	+	24º00'37.45'' N, 104º07'31.73 W
CD-TO	2015	*Amaranthaceae*	*Amaranthus spp*		26º04'52.61'' N, 102º55'30.01'' W
CD-LG	2015	*Asteraceae*	*Artemisia absitium*		25º29'28.04'' N, 103º41'12.80 W
CD-TL	2015	*Asteraceae*	*Artemisia absitium*		25º47'14.25'' N, 103º21'10.37'' W
CD-TO	2015	*Asteraceae*	*Bahia absinthifolia*		25º32'46.79'' N, 103º16'55.40'' W
CD-TO	2015	*Asteraceae*	*Bahia absinthifolia*		25º32'46.79'' N, 103º16'55.40'' W
CD-LG	2015	*Asteraceae*	*Helianthus spp*		25º29'28.04'' N, 103º41'12.80 W
CD-LG	2015	*Asteraceae*	*Helianthus spp*		25º29'28.04'' N, 103º41'12.80 W
CD-TO	2015	*Asteraceae*	*Helianthus spp*		25º33'21.45'' N, 103º22'17.73'' W
CD-TL	2015	*Asteraceae*	*Sonchus oleraceus*		25º47'14.25'' N, 103º21'10.37'' W
CD-TL	2015	*Asteraceae*	*Sonchus oleraceus*		25º47'14.25'' N, 103º21'10.37'' W
CD-TL	2015	*Asteraceae*	*Sonchus oleraceus*		25º47'14.25'' N, 103º21'10.37'' W
CD-TL	2015	*Asteraceae*	*Sonchus oleraceus*		25º47'14.25'' N, 103º21'10.37'' W
CD-TL	2015	*Asteraceae*	*Sonchus oleraceus*		25º47'14.25'' N, 103º21'10.37'' W
CD-TO	2015	*Asteraceae*	*Xanthium echinatum*		25º32'46.79'' N, 103º16'55.40'' W
CD-TO	2015	*Asteraceae*	*Xanthium echinatum*		25º32'46.79'' N, 103º16'55.40'' W
CD-TO	2015	*Asteraceae*	*Xanthium echinatum*		25º32'46.79'' N, 103º16'55.40'' W
CD-LG	2015	*Malvaceae*	*Sida acuta*	+	25º29'28.04'' N, 103º41'12.80 W
CD-LG	2015	*Malvaceae*	*Sida rombifolia*	+	25º29'28.04'' N, 103º41'12.80 W

CD-LG	2015	*Malvaceae*	*Sida rombifolia*	+	25º29'28.04'' N, 103º41'12.80 W
CD-TL	2015	*Malvaceae*	*Sida rombifolia*	+	25º47'14.25'' N, 103º21'10.37'' W
CD-TL	2015	*Malvaceae*	*Sida rombifolia*	+	25º47'14.25'' N, 103º21'10.37'' W
CD-TL	2015	*Malvaceae*	*Sida rombifolia*	+	25º47'14.25'' N, 103º21'10.37'' W
CD-TO	2015	*Malvaceae*	*Sphaeralcea angustifolia*	+	25º32'46.79'' N, 103º16'55.40'' W
CD-TO	2015	*Malvaceae*	*Sphaeralcea angustifolia*	+	25º32'46.79'' N, 103º16'55.40'' W
CD-TO	2015	*Malvaceae*	*Sphaeralcea angustifolia*	+	26º04'52.61'' N, 102º55'30.01'' W
CD-LG	2015	*Nyctaginaceae*	*Boerhavia coccinea*	+	25º29'28.04'' N, 103º41'12.80 W
CD-PO	2016	*Portulacaceae*	*Portulaca oleraceae*		24º00'37.45'' N, 104º07'31.73 W
CD-PO	2016	*Portulacaceae*	*Portulaca oleraceae*		24º00'37.45'' N, 104º07'31.73 W
CD-PO	2016	*Portulacaceae*	*Portulaca oleraceae*		24º00'37.45'' N, 104º07'31.73 W
CD-PO	2016	*Portulacaceae*	*Portulaca oleraceae*		24º00'37.45'' N, 104º07'31.73 W
CD-PO	2016	*Portulacaceae*	*Portulaca oleraceae*		24º00'37.45'' N, 104º07'31.73 W
CD-PO	2016	*Portulacaceae*	*Portulaca oleraceae*		24º00'37.45'' N, 104º07'31.73 W
CD-LG	2015	*Solanaceae*	*Datura stramonium*	+	25º29'28.04'' N, 103º41'12.80 W
CD-TO	2015	*Solanaceae*	*Datura stramonium*	+	25º33'21.45'' N, 103º22'17.73'' W
CD-TO	2015	*Solanaceae*	*Nicotiana glauca*	+	25º32'46.79'' N, 103º16'55.40'' W
CD-TO	2015	*Solanaceae*	*Nicotiana glauca*	+	25º32'46.79'' N, 103º16'55.40'' W
CD-TO	2015	*Solanaceae*	*Nicotiana glauca*	+	26º04'52.61'' N, 102º55'30.01'' W
CD-PO	2016	*Solanaceae*	*Solanum elaeagnifolium*	+	24º00'37.45'' N, 104º07'31.73 W
CD-PO	2016	*Solanaceae*	*Solanum elaeagnifolium*	+	24º00'37.45'' N, 104º07'31.73 W
CD-TL	2015	*Solanaceae*	*Solanum elaeagnifolium*	+	25º47'14.25'' N, 103º21'10.37'' W
CD-TO	2015	*Solanaceae*	*Solanum elaeagnifolium*	+	25º32'46.79'' N, 103º16'55.40'' W
CD-TO	2015	*Solanaceae*	*Solanum elaeagnifolium*	+	25º32'46.79'' N, 103º16'55.40'' W
CD-TO	2015	*Solanaceae*	*Solanum elaeagnifolium*	+	25º32'46.79'' N, 103º16'55.40'' W
CD-PO	2016	*Solanaceae*	*Solanum rostrarum*	+	24º00'37.45'' N, 104º07'31.73 W
CD-PO	2016	*Solanaceae*	*Solanum rostrarum*	+	24º00'37.45'' N, 104º07'31.73 W
CD-PO	2016	*Solanaceae*	*Solanum rostrarum*	+	24º00'37.45'' N, 104º07'31.73 W

[1] Designations for sampling area. Sampling areas were divided in five regions located in different biogeographic zones: Baja California (BC), Sonora (SO), Sinaloa (SI), Colima-Nayarit (CN), and Coahulia-Durango (CD). BC-SQ: Baja California, San Quintin; BC-EN: Baja California, Ensenada; BC-ME: Baja California, Mexicali; SO-OB: Sonora, Obregon; SO-NA: Sonora, Navojoa; SO-HU: Sonora, Huatabampo; SO-RC: Sonora, Rio Colorado; SI-GV: Sinaloa, Guasave; SI-SL: Sinaloa, Sinaloa de Leyva; SI-MO: Sinaloa, Mocorito; SI-PC: Sinaloa, Playa Ceuta; SI-CO: Sinaloa, Concordia; SI-AC: Sinaloa, Agua Caliente; SI-RO: Sinaloa, El Rosario; CN-SO: Colima-Nayarit, Santa Maria del Oro; CN-TE: Colima-Nayarit, Tecoman; CD-TL: Coahuila-Durango, Tlahualilo; CD-LG: Coahuila-Durango, La Goma; CD-TO: Coahuila-Durango, Torreon; CD-PO: Coahuila-Durango, Poanas.

[2] PCR begomovirus detection. Universal primers to detect both monopartite and bipartite members of begmomovirus genera (Supplementary Table S3).

(+). Positive begomovirus detection.

Supplementary Table S2. Geminivirus signatures obtained by *de novo* assembly from the metagenomics study in plants (agro-ecological interface) from five regions in north-pacific Mexico. Geminivirus-related reads for each NGS library were used for *de novo* assembly and generation of signatures. Contigs aligments of 100-299 bp in length were selected regardless whether one or both viral components were detected. A total of 59 different geminivirus species were detected, 33 and 26 from cultivated and from non-cultivated plant-adapted species, respectively.

**Host adapted**	**Virus acronym**[1]	**Plant Family of first detection**	Geminivirus-signatures of DNA-A/DNA-B[2]				
			**Baja California**	**Sonora**	**Sinaloa**	**Colima-Nayarit**	**Coahuila-Durango**
**Crops**	CdTV	*Solanaceae*	ND[3]	ND	93.2/92.9 (AF101476.1/EU339940.1) (148/171)	86.5/ND (DQ885456.1/ND) (178/ND)	ND
	ToYLDV		ND	ND	88.1/87.2 (HE806440.1/HE806441.1) (160/156)	ND	ND
	ToYVSV		ND	88.5/ND (KC136339.1/ND) (157/ND)	ND	ND	ND
	ToLV		ND	ND	88/ND (KM926624.1/ND) (164/ND)	ND	ND
	ToLDeV		ND	ND	ND	87.6/ND (JX501506.1/ND) (186/ND)	ND
	ToMoLCV		ND	ND	ND	87.6/78.2 (KX896414.1/JF803264.1) (227/115)	ND
	ToMoTaV		ND	ND	ND/81.6 (ND/AF012301.1) (ND/174)	ND	ND
	ToMoV		ND	ND	94.1/ND (DQ865248.1/ND) (103/ND)	ND	ND
	ToGMoV		ND	ND	ND/85 (ND/DQ406674.1) (ND/161)	ND	ND
	ToCmMV		ND	ND	ND	ND/83.9 (ND/KT203559.1) (ND/149)	ND
	ToClLDV		ND	87.9/ND (JN241632.1/ND) (132/ND)	ND	ND/84 (ND/HQ201953.1) (ND/132)	ND
	ToYLDV		ND	ND	ND	81.4/ND (KU232891.1/ND) (124/ND)	ND
	ToYMLCV		ND	ND	ND	82.2/ND	ND

						(AY508993.2/ND) (135/ND)	
	TRYLCV		ND	ND	ND	84.1/79.2 (KC132844.1/JN381820.1) (101/149)	ND
	ToLCCNV		ND	ND	ND	90.2/NA[4] (KU980922.1/NA) (113/NA)	ND
	ToLCSDV		ND	ND	ND	90.8/NA (HG941648.1/NA) (207/NA)	ND
	TbLCYnV		ND	ND	93.4/NA (AJ971267.1/NA) (152/NA)	ND	ND
	TbLCCuV		ND	ND	93.4/ND (KU562963.1/ND) (183/ND)	ND	ND
	TbMoLCV		ND	ND	91/ND (FM160943.1/ND) (123/ND)	ND	ND
	TbLRV		ND	ND	96.1/ND (AJ488768.2/ND) (155/ND)	ND	ND
	DaLDV		ND	ND	86.4/93 (JN848773.1/JN848774.1) (111/129)	ND	ND
	CLCrV	*Malvaceae*	ND	ND/98 (ND/AY742221.1) (ND/251)	ND/87.2 (ND/AF480941.1) (ND/157)	ND	ND
	CLCuV		ND	ND	87.4/NA (AJ228570.1/NA) (119/NA)	ND	ND
	CoChSpV		ND	ND	ND	ND/89.8 (ND/KF358471.1) (ND/138)	ND
	AbMBV		ND	ND	96.1/ND (LN611622.1/ND) (103/ND)	90/79.8 (JF694482.1/ JF694483.1) (110/129)	ND
	CabLCJV	*Brassicaceae*	ND	ND	ND	92.7/ND (DQ178614.1/ND) (124/ND)	ND
	CBSMV	*Fabaceae*	ND	ND/88.3 (ND/KX096982.1) (ND/197)	ND	89.8/89.6 (KX011476.1/KX096982.1) (148/155)	ND
	BGYMV		ND	ND	ND	81.1/89.1 (M10080.1/KU145406.1)	ND

						(154/157)	
	SbBMV		ND	91.4/ND (EF016486.1/ND) (209/ND)	ND	ND	ND
	MCLCuV	*Cucurbitaceae*	ND	ND	ND	95/82.9 (KF670624.1/KF670631.1) (121/123)	ND
	SYMMoV		ND	ND	ND	86.9/88.4 (KC153490.1/KC153491.1) (107/147)	ND
	JMV	*Euphorbiaceae*	ND	ND	90.3/88.9 (KF998097.1/KF998098.1) (155/153)	ND	ND
	PaLCuV	*Caricaceae*	ND	ND	ND	91.8/NA (KT266873.1/NA) (122/NA)	ND
**Non-cultivated plants**	SiMBoV	*Malvaceae*	ND	ND/89.7 (ND/HM585444.1) (ND/107)	ND	ND	ND
	SimMV		ND	87.4/87.3 (KX348159.1/KC706532.1) (278/173)	92.5/81.3 (KC706535.1/EU908734.1) (121/161)	90.2/87.3 (HM357459.3/FN557523.1) (144/142)	ND
	SiGMV		ND	ND/80.5 (ND/AJ250731.1) (ND/216)	94.7/94 (Y11097.1/Y11098.1) (152/252)	96.1/80.5 (KT099120.1/KT099161.1) (154/159)	ND
	SiGMBuV		ND	ND	93.5/ND (DQ665864.1/ND) (154/ND)	ND	ND
	SiGYVV		ND	ND	91.3/86.3 (KT879825.1/KT879827.1) (161/117)	ND	ND
	SiAMV		ND	ND	ND	ND/87.7 (ND/KX691415.1) (ND/114)	ND
	SiCVV		ND	ND	ND	76.6/78.75 (KX691405.1/KX691413.1) (177/160)	ND
	SiCMV		ND	ND	88.6/ND (JX871370.1/ND) (149/ND)	79.6/78.7 (KX691405.1/KX691413.1) (177/160)	ND
	SiYNV		ND	ND	ND	84.5/ND (JX871376.1/ND) (188/ND)	87/ND (JX871376.1/ND) (232/ND)
	SiYVV		ND	ND	80.8/ND (Y11099.1/ND) (198/ND)	ND	ND

	PavYMV		ND	ND	ND	ND/83.8 (ND/KT948788.1) (ND/142)	ND
	TrYMV		ND	ND/87.2 (ND/ KU131590.1) (ND/212)	ND	ND	ND
	RhRGMV	*Fabaceae*	ND	ND	ND	ND/77.2 (ND/HM236371.1) (ND/176)	ND
	MacGMV		ND	ND	93.6/87.5 (EU158096.1/DQ979834.1) (158/128)	ND	ND
	MacCMV		ND	ND	ND	ND/82.3 (ND/KX691412.1) (ND/119)	ND
	MacYMV		ND	ND	ND	76.4/86.4 (AJ344452.1/EF585289.1) (221/118)	ND
	CaGMV		ND	89/ND (AF439402.1/ND) (173/ND)	94.7/ND (AF439402.1/ND) (153/ND)	90.5/ND (AF439402.1/ND) (148/ND)	ND
	DesLDV		ND	ND	92.2/ND (DQ318930.1/ND) (155/ND)	ND	ND
	DiYMoV	*Euphorbiaceae*	ND	ND	ND	ND/86.4 (ND/AF170101.1) (ND/155)	ND
	ChLCV	*Amaranthaceae*	ND	ND	ND	ND/89.7 (ND/KJ826528.1) (ND/117)	ND
	AYVV	*Astaraceae*	ND	ND	ND	87.7/NA (KX759647.1/NA) (285/NA)	ND
	CleLCrV	*Cleomaceae*	ND	ND/84 (ND/JF694460.1) (ND/144)	ND	ND	ND
	MerMV	*Convolvulaceae*	ND	ND	87.5/ND (KT693180.1/ND) (112/ND)	ND	ND
	LaYVV	*Linderniaceae*	ND	ND	ND	90/ND (DQ641701.1/ND) (121/ND)	ND
	PouGMV	*Urticaceae*	ND	ND	ND	91.22/ND (KU358528.1/ND) (114/ND)	ND
	StaLCuV	*Verbenaceae*	ND	ND	82.7/ND (AJ564743.2/ND)	ND	ND

					(151/ND)		

[1] Virus acronyms:

Monopartite Geminiviruses: *Cotton leaf curl virus* (CLCuV), *Tobacco leaf curl Yunnan virus* (TbLCYnV), *Tomato leaf curl China virus* (ToLCCNV), *Tomato leaf curl Sudan virus* (ToLCSDV).

Bipartite Geminiviruses: *Abutilon mosaic virus* (AbMV), *Ageratum yellow vein virus* (AYVV), Bean golden yellow mosaic virus (BGYMV), *Cabbage leaf curl Jamaica virus* (CabLCJV), *Calopogonium golden mosaic virus* (CaGMV), *Common bean severe mosaic virus* (CBSMV), *Chino del tomate virus* (CdTV), *Chenopodium leaf curl virus* (ChLCV), *Cotton leaf crumple virus* (CLCrV), *Cotton leaf curl virus* (CLCuV), *Cleome leaf crumple virus* (CleLCrV), *Cotton chlorotic spot virus* (CoChSPV), *Datura leaf distortion virus* (DaLDV), *Desmodium leaf distortion virus* (DesLDV), *Dicliptera yellow mottle virus* (DiYMoV), *Jatropha mosaic virus* (JMV), *Lindernia anagallis yellow vein virus* (LaYVV), *Macroptilium common mosaic virus* (MacCMV), *Macroptilum golden mosaic virus* (MacGMV), *Macroptilium yellow mosaic virus* (MacYMV), *Melon chlorotic leaf curl virus* (MCLCuV), *Merremia mosaic virus* (MerMV), *Papaya leaf curl virus* (PaLCuV), *Pavonia yellow mosaic virus* (PavYMV), *Pouzolzia golden mosaic virus* (PouGMV), *Rhynchosia rugose golden mosaic virus* (RhRGMV), *Soybean blistering mosaic virus* (SbBMV), *Sida angular mosaic virus* (SiAMV), *Sida common mosaic virus* (SiCMV), *Sida chlorotic vein virus* (SiCVV), *Sida golden mosaic Backup virus* (SiGMBuV), *Sida golden mosaic virus* (SiGMV), *Sida golden yellow vein virus* (SiGYVV), *Sida mosaic Bolivia virus* (SiMBoV), *Sida micrantha mosaic virus* (SimMV), Sida yellow net virus (SiYNV), *Sida yellow vein virus* (SiYVV), *Stachytarpheta leaf curl virus* (StaLCuV), *Squash yellow mild mottle virus* (SYMMoV), *Tobacco leaf curl Cuba virus* (TbLCCUV), *Tobacco leaf rugose virus* (TbLRV), *Tobacco mottle leaf curl virus* (TbMoLCV), *Tomato chlorotic leaf distortion virus* (ToClLDV), *Tomato common mosaic virus* (ToCmMV), *Tomato golden mottle virus* (ToGMoV), *Tomato leaf deformation virus* (ToLDeV),*Tomato latent virus* (ToLV), *Tomato mottle leaf curl virus* (ToMoLCV), *Tomato mottle Taino virus* (ToMoTaV), *Tomato mottle virus* (ToMoV), *Tomato yellow leaf distortion virus* (ToYLDV), *Tomato yellow margin leaf curl virus* (ToYMLCV), *Tomato yellow vein streak virus* (ToYVSV), *Tomato rugose yellow leaf curl virus* (TRYLCV), *Triumfetta yellow mosaic virus* (TrYMV).

[2]Best match in %, accession numbers, and contig length aligned.

[3] ND: No detected.

[4] NA: Not applicable

Supplementary Table S3. List of PCR primers used in the present work

Virus	Primer name	Primer sequence 5' to 3'	Expected amplicon lenght (pb)	Reference
Begomovirus[1]	DGR-SAR	GAGTCTAGATGCTGACCTCCTCTAGCWGATCTGC	950 pb-1150 pb	Mauricio Castillo et al., 2007.
	CP-70	CACGGATCCGATTGRACCTTACANGGNCCTTCACA		De la Torre-Almaraz et al., 2006
TYLCV	qPCR-TYLCV-F	GAAGGCTGAACTTCGACAGC	171	Rodriguez-Negrete et al., 2014
	qPCR-TYLCV-R	GGACTTTACATGGGCCTTCAC		
PHYVV	qPCR-PHYVV-F	GGCGATACCGTAGAATGGGGAGAA	158	Morales-Aguilar et al., 2019
	qPCR-PHYVV-R	TGAAGGAAGAAATGCTGGGGTTGT		
RhGMV/RhGMSV[2]	qPCR-RhGMV-F	CTTGGTACCCCTATGGATTTTGGC	146	This work

SiMSiV	qPCR-RhGMV-F<br>qPCR-SiMSV-F<br>qPCR-SiMSV-R	CGTTGCTGGCATACTGTCCACC<br>TTGGCAAGATATGGATGGATGA<br>CAGTGCTGGGCTCGTTGTCG	147	This work

[1] Universal primer set designed to detect both monopartite and bipartite members of begmomovirus genera.

[2] Primer set designed to detect simultaneously both begomovirus species (RhGMV/RhGMSV)

Virus acronyms. Pepper huasteco yellow vein virus (PHYVV), *Tomato yellow leaf virus* (TYLCV), *Sida mosaic Sinaloa virus* (SiMSiV), *Rhynchosia golden mosaic virus* (RhGMV), *Rhynchosia golden mosaic Sinaloa virus* (RhGMSV).
